# Adipose‐specific ATGL ablation reduces burn injury‐induced metabolic derangements in mice

**DOI:** 10.1002/ctm2.417

**Published:** 2021-06-06

**Authors:** Supreet Kaur, Christopher Auger, Dalia Barayan, Priyal Shah, Anna Matveev, Carly M. Knuth, Thurl E. Harris, Marc G. Jeschke

**Affiliations:** ^1^ Ross Tilley Burn Centre Sunnybrook Health Sciences Centre Toronto Ontario Canada; ^2^ Institute of Medical Sciences University of Toronto Toronto Ontario Canada; ^3^ Department of Pharmacology University of Virginia School of Medicine Charlottesville VA USA

**Keywords:** adipose triglyceride lipase, browning, burns, FGF21, mitochondria, trauma, uncoupling

## Abstract

Hypermetabolism following severe burn injuries is associated with adipocyte dysfunction, elevated beige adipocyte formation, and increased energy expenditure. The resulting catabolism of adipose leads to detrimental sequelae such as fatty liver, increased risk of infections, sepsis, and even death. While the phenomenon of pathological white adipose tissue (WAT) browning is well‐documented in cachexia and burn models, the molecular mechanisms are essentially unknown. Here, we report that adipose triglyceride lipase (ATGL) plays a central role in burn‐induced WAT dysfunction and systemic outcomes. Targeting adipose‐specific ATGL in a murine (AKO) model resulted in diminished browning, decreased circulating fatty acids, and mitigation of burn‐induced hepatomegaly. To assess the clinical applicability of targeting ATGL, we demonstrate that the selective ATGL inhibitor atglistatin mimics the AKO results, suggesting a path forward for improving patient outcomes.

## Graphical abstract

- A severe burn injury and its associated hypermetabolic response induces adipocyte dysfunction, fat catabolism, elevated fatty acids in the circulation and hepatic steatosis.
- Adipose triglyceride lipase (ATGL) plays a central role in adipose lipolysis and lipid metabolism.
- Targeting adipose‐specific ATGL post‐burn injury diminishes WAT browning, reduces fatty acids in the circulation and protects against liver dysfunction.

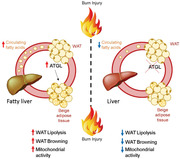

## 1 INTRODUCTION

Hypermetabolism, characterized by substantial and persistent increases in resting energy expenditure (REE), is a hallmark of a severe burn, trauma, and brain injuries.^1^, ^2^ It is also present in patients suffering from infectious diseases, such as human immunodeficiency virus and advanced cancers (cachexia).^2^, ^3^ This increase in REE (>110% of predicted REE) perpetuates a catabolic state characterized by supraphysiological nutritional requirements, weight loss, muscle atrophy, and the breakdown of adipose depots.^1^, ^4^ Systemically, these changes culminate in organ dysfunction and failure, as demonstrated by the increased incidence of acute kidney injury, heart, and liver disease seen in intensive care unit patients.^5^ Although hypermetabolism is present during these aforementioned disease processes, the sheer magnitude and duration of the hypermetabolic response are unique in burn patients. REE can reach levels up to 220%–250% of predicted and also persist for years after the initiating event, inducing a cascade of devastating sequelae.^6^ Indeed, the loss of body mass leads to poor wound healing and increased risk of infections, sepsis, and ultimately death, pinpointing hypermetabolism as the primary mediator of patient mortality.^1^ Although the pathophysiology linked to hypermetabolic responses has been documented, the mechanisms are essentially unknown.

A critical failure of the research and healthcare community working with patients suffering from traumatic injuries and hypermetabolism is overlooking the role of adipose tissue as a contributor to poor outcomes. Once considered a mere storage depot, white adipose tissue (WAT) is now appreciated as a dynamic endocrine organ with roles in initiating and sustaining systemic dysfunction. This may occur via the release of adipokines, such as leptin, or the induction of thermogenic brown adipocytes within WAT (“beige” or “brite” cells).^7^, ^8^ While often praised for its potential benefits in metabolic diseases such as obesity or diabetes, browning and the activation of uncoupling protein 1 (UCP‐1) in hypermetabolic conditions such as in burns and cancer cachexia appear to be detrimental and exhaust body mass reserves.^9^ For instance, the browning of subcutaneous WAT (scWAT) is an early systemic event in cancer cachexia pathophysiology recognized to contribute to increased REE and adipose breakdown; however, the browning response in cachexia model is debatable.^9^ Following a severe burn, WAT adopts an inflammatory state associated with NLRP3 activation, browning, and increased lipolysis with an altered circulating lipidomic profile.^10^, ^11^ Recently, it has been demonstrated that specific pharmacologic interventions can reduce adipose browning and systemic dysfunction, further cementing the role of adipose tissue in perpetuating poor outcomes for burn patients.^12^, ^13^ To that effect, the underlying mechanisms of pathological browning and changes to adipose physiology remain poorly understood.

Adipose triglyceride lipase (ATGL) is a key triacylglycerol (TAG) lipase responsible for the breakdown of fat stores into free fatty acids (FFA) and glycerol. While it was thought that ATGL is required as a source of FFA for UCP‐1 activation during interscapular brown adipose tissue (BAT) cold‐induced thermogenesis, this has recently been explored.^14^ However, the role of this enzyme and FFA release in scWAT browning is not well understood, particularly in pathological contexts. Burn patients suffer from a chronic increase in β‐adrenergic signaling mediated by catecholamines, known inducers of endoplasmic reticulum stress and lipolysis.^15^ While the initial spike in epinephrine and norepinephrine levels is of benefit to fuel the fight‐or‐flight response, the perpetual stimulation of lipolysis induces an extreme loss of WAT mass that is only partially mitigated by β‐blockade.^16^ To that effect, we have turned our attention toward the study of lipolytic enzymes as effectors of poor outcomes post‐burn and the possibility of therapeutically targeting lipolysis in hypermetabolism to limit FFA‐mediated damage to lean muscle and internal organs.

This study aimed to assess the physiological relevance of adipose ATGL in burn‐induced systemic dysfunction. To do so, adipose‐specific ATGL knockout (AKO) and ATGL floxed (WT) mice were subjected to a 30% total body surface area (TBSA) thermal injury and then select biomarkers studied at 1‐week post‐burn, a time point that translates to months in human patients. Atglistatin, a recently described inhibitor of murine ATGL, was administered to determine the therapeutic potential of ATGL targeting.^17^ In parallel, adipose samples from consenting patients which are routinely discarded during early excision of the burn wound were analyzed for thermogenic and lipolytic markers to demonstrate their relevance following the initial injury. Based on our findings, we suggest that burn injury induces a vicious cycle of ATGL‐mediated lipolysis and thermogenesis followed by FFA‐stimulated fibroblast growth factor 21 (FGF21) release, which further perpetuates adipose dysfunction. Additionally, we have elucidated the clinical importance of these findings by assessing the role of the selective inhibitor atglistatin, which demonstrates the beneficial potential of targeting ATGL on WAT physiology and liver function in hypermetabolic conditions.

## 2 RESULTS

### 2.1 Burns upregulate the expression of ATGL, UCP1, and FGF21 in adipose tissue

**FIGURE 1:**
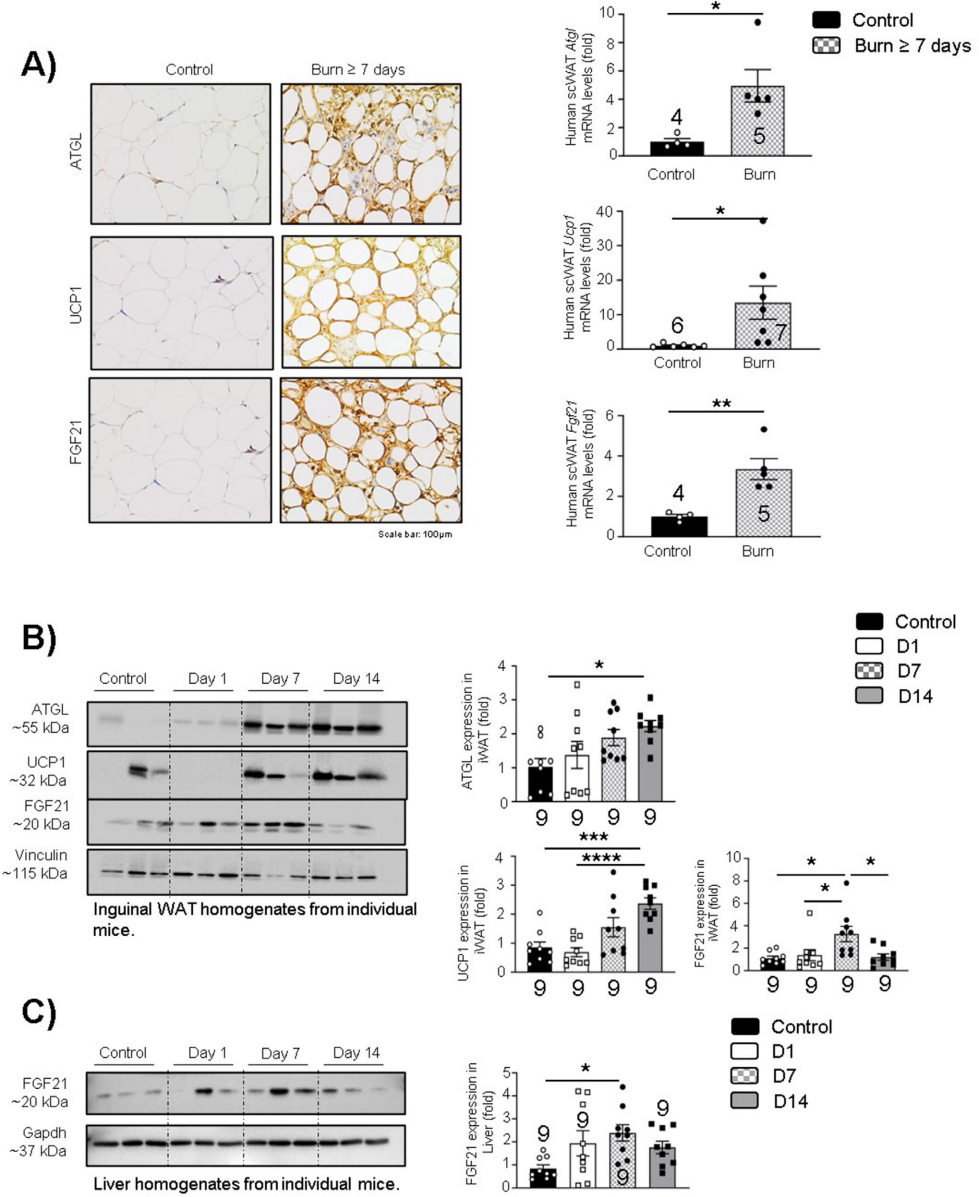
Alterations in ATGL, UCP1, and FGF21 expression in hypermetabolic conditions: (A) Immunohistochemistry and quantitative real‐time PCR in fat samples obtained from consented normal versus burn (≥7 days) human patients and assessed for *Atgl, Ucp1*, and *Fgf21* expression. (B) Western blot analysis and quantification of murine WAT biopsies collected on days 1, 7, and 14 and analyzed for ATGL, UCP1, and FGF21 protein expression. (C) Western blot analysis and quantification of murine liver biopsies collected on days 1, 7, and 14 and analyzed for FGF21 protein expression. The results displayed are the average and SEM analyzed in the specified number of mice samples. Statistical significance was assessed using one‐way ANOVA as appropriate

To assess the contribution of WAT lipolysis following burn injury, we analyzed the expression of key markers such as ATGL, UCP1, and FGF21 in human and murine adipose tissue obtained during surgical procedures (Table S1) by immunohistochemistry (IHC), western blot, and gene expression studies. *Atgl*, *Ucp1*, and *Fgf21* expressions were increased in human burn patient scWAT, suggesting their adipose tissue undergoes browning as well as a link between WAT lipolysis and thermogenesis in this setting (Figure 1A). Moreover, a time‐course study in inguinal WAT (iWAT) samples from a murine thermal injury model showed similar results. Murine ATGL and UCP1 expressions increase in a time‐dependent manner in iWAT, demonstrating no change on day 1, a trending increase on day 7 and enhanced expression on day 14 respectively after burn (Figure 1B). FGF21 expression in iWAT peaks on day 7 but goes back to normal on day 14 (Figure 1B). Similar results were observed in murine hepatic FGF21 expression (Figure 1C), indicating that this protein might be acting as a trigger in liver‐WAT cross‐talk post‐burn injury. These findings suggest an interplay between alterations in ATGL, UCP1, and FGF21 expression post‐burn injury and raise the possibility that ATGL plays a central role in this loop. Therefore, we elected to investigate the role of ATGL in the activation of WAT lipolysis and browning using an adipose‐specific ATGL KO mouse model in hypermetabolic conditions.

### 2.2 Adipose‐specific ATGL deletion reduces WAT lipolysis and systemic fat circulation

**FIGURE 2:**
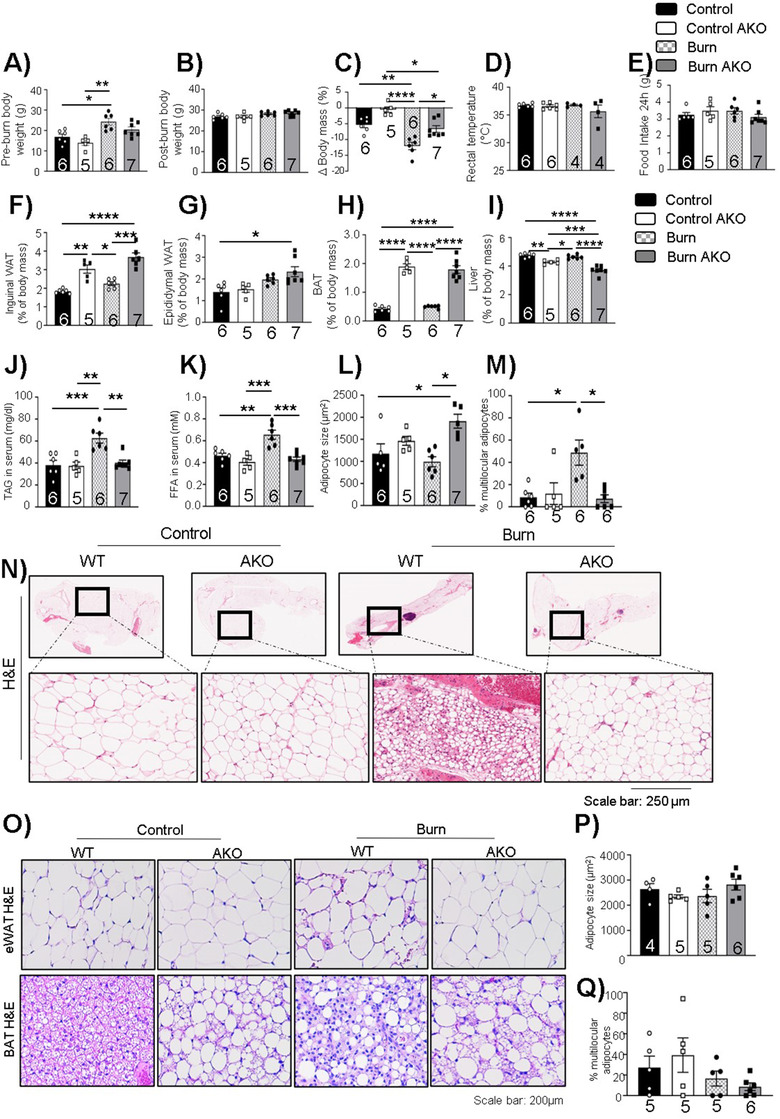
Adipose‐specific ATGL deletion impact on body weight and adipose tissue post‐burn injury: Twelve‐week‐old ATGL floxed and knockout mice were treated with 30%TBSA injury and monitored daily for 7 days. (A) Pre‐burn body weight. (B) Post‐burn body weight. (C) Change in body mass on day 7 in comparison to day 0. (D) Rectal temperature at day 7 of individual mice. (E) Food intake (24 h) at day 7 of individual mice. (F) Inguinal white adipose tissue (iWAT). (G) Epididymal white adipose tissue (eWAT). (H) Brown adipose tissue (BAT), and (I) Liver normalized to the body weight of individual mice. (J) Triacylglycerol (TAG) content in serum samples. (K) Free fatty acid levels in serum samples. (L) Adipocyte size of adipocytes in iWAT. (M) Percentage of normalized multilocular adipocytes normalized to total number of adipocytes in sections of iWAT. (N) Hematoxylin and eosin (H&E) staining (zoom in and zoom out images of whole fat pad) in WAT samples. (O) Hematoxylin staining of epididymal and brown adipose tissue. (P) Adipocyte size of adipocytes in eWAT. (Q) Percentage of normalized multilocular adipocytes normalized to total number of adipocytes in sections of epididymal adipose tissue. The results displayed are the average and SEM analyzed in the specified number of mice samples. Statistical significance was assessed using two‐way ANOVA as appropriate

To understand the specific contribution of ATGL in WAT post‐burn injury, we used an adipose‐specific ATGL KO adult murine model (AKO) that lacks this enzyme in all fat pads while ATGL floxed mice served as wild type (WT) controls. A severe burn injury results in the loss of body mass in burn‐injured mice (∼10%), which was found to be reverted in AKO mice, suggesting that ATGL deletion protects against loss of body mass post‐burn injury (Figures 2A‐2C). However, no changes were observed in the rectal temperature (Figure 2D) and food intake (Figure 2E) in the test groups. Adipose‐specific ATGL deletion resulted in increased iWAT mass for AKO mice, an impact independent of burn injury (Figure 2F). Similar impact of adipose‐specific ATGL deletion was observed on eWAT (Figure 2G) and BAT mass (Figure 2H) for AKO mice. Furthermore, adipose‐specific ATGL deletion resulted in reduced liver mass in burn‐injured AKO mice in comparison to burn control mice (Figure 2I), suggesting that burn AKO mice had reduced systemic fat in the circulation that protected against hepatomegaly post‐burn injury. To test this hypothesis, we assessed TAG and FFA in serum samples. Indeed, while the burn injury WT group showed elevated levels of TAG (Figure 2J) and FFA (Figure 2K) in systemic circulation, burn AKO mice had levels comparable to WT controls, suggesting no changes in systemic fat circulation in response to burn injury as a result of ATGL deletion in murine fat pads. Furthermore, hematoxylin and eosin (H&E) staining was performed on whole fat pad to assess the impact of ATGL deletion on adipocyte size and structure. Interestingly, burn AKO murine adipocytes showed increased adipocyte size (Figure 2L) and a low percentage of multilocular adipocytes (Figures 2M and 2N) in comparison to the burn‐alone group. A similar trend was observed in eWAT (Figures 2O‐2Q). AKO mice also seem to have larger adipocytes in BAT in comparison to WT mice (Figure 2O), an impact independent of burn injury. Due to ATGL deletion, these mice have an enhanced tendency to accumulate fat.

**FIGURE 3:**
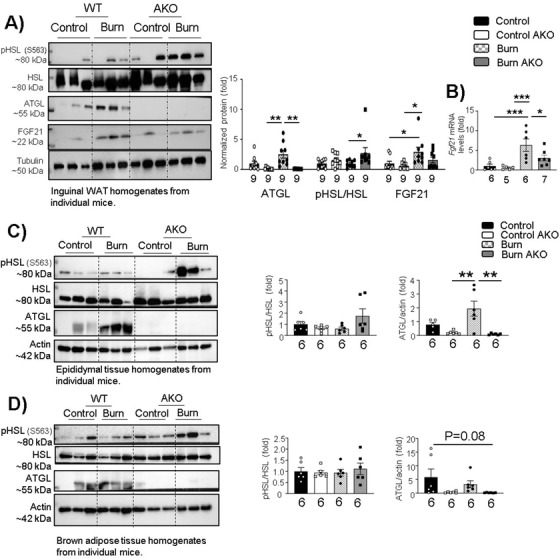
Adipose‐specific ATGL deletion impact on adipose tissue lipolysis post‐burn injury: Twelve‐week‐old ATGL floxed and knockout mice were administered a 30%TBSA thermal injury and monitored daily for 7 days. (A) Western blot analysis and quantification of the expression of targeted proteins (p‐HSL ser 563, HSL, ATGL, FGF21, and tubulin) in WAT. (B) Quantitative PCR analysis of *Fgf21* expression in WAT. Western blot analysis and quantification of lipolysis markers (pHSL, HSL, and ATGL) in (C) epididymal adipose tissue and (D) brown adipose tissue. The results displayed are the average and SEM analyzed in the specified number of mice samples. Statistical significance was assessed using two‐way ANOVA as appropriate

Next, we wanted to assess the impact of adipose‐specific ATGL deletion post‐burn injury on ATGL, phospho‐HSL (Serine 563), and total HSL (hormone‐sensitive lipase). The latter converts DAG (diglyceride) into MAG (monoglyceride) and has reduced substrate specificity toward TAG in comparison to ATGL.^18^ As expected, ATGL expression was wiped out in all fat pads (Figures 3A, 3C, and 3D). Phospho‐HSL (Ser 563; normalized) levels were found elevated in iWAT samples in burn AKO mice (Figure 3A). We also elected to study the hepatokine FGF21 in iWAT samples. We observed that FGF21 protein and gene expressions were elevated in iWAT samples in burn WT mice (Figures 3A and 3B), but this impact was reduced in burn AKO mice, suggesting a link between ATGL deletion in iWAT and FGF21 expression in post‐burn hypermetabolism.

### 2.3 Adipose‐specific ATGL deletion suppresses mitochondrial activity and prevents browning in WAT

**FIGURE 4:**
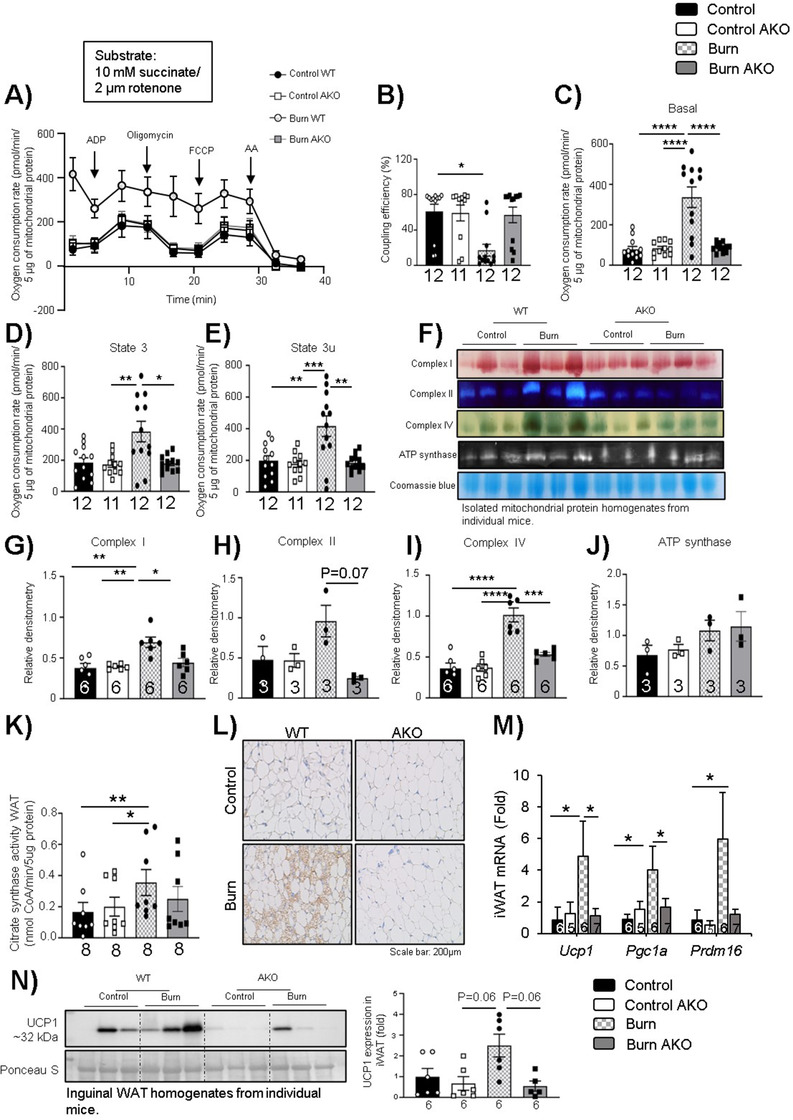
Adipose‐specific ATGL deletion suppresses mitochondrial activity and prevents browning in WAT post‐burn injury: Twelve‐week‐old ATGL floxed and knockout mice were treated with 30%TBSA injury and monitored daily for 7 days. (A) Mitochondria respiration profiles of isolated WAT mitochondria from control (black), control AKO (white), burn (light grey), and burn AKO (dark grey). (B) Coupling efficiency (%) measured by the Seahorse stress test report generator. Respiration paramateres (C) Basal, (D) state 3, (E) state 3u in isolated mitochondria as measured via Seahorse assay. (F‐J) Isolated mitochondrial protein was run and assessed in a gradient gel via BN‐PAGE, and activity assays were performed for complexes such as I, II, IV, and ATP synthase. (K) Citrate synthase activity. (L) Immunohistochemistry for UCP1 expression. (M) Quantitative PCR analysis of browning markers (*Ucp1*, *Pgc1a*, and *Prdm16*) and (N) Western blot analysis of UCP1 expression normalized to Ponceau S as loading control in WAT. The results displayed are the average and SEM analyzed in the specified number of mice samples. Statistical significance was assessed using two‐way ANOVA tests as appropriate

A major characteristic of burn‐induced hypermetabolism is enhanced mitochondrial activity accompanied by thermogenesis in WAT, physiological changes termed pathological browning, and leading to enhanced REE.^1^, ^12^, ^19^ To that effect, we assessed the impact of adipose‐specific ATGL ablation on WAT mitochondrial activity when challenged with burn injury. Fresh WAT was collected from mice following euthanasia and immediately homogenized for mitochondrial activity assays (Seahorse XF96 extracellular flux). As expected, burn WT mice showed enhanced mitochondrial activity and reduced coupling efficiency on day 7 (Figures 4A and 4B). Intriguingly, ATGL ablation reduces mitochondrial activity in iWAT, with respiration curves and coupling efficiency similar to controls (Figures 4A and 4B). This reduction in mitochondrial oxygen consumption in burn AKO mice versus burn WT is evident in all states of respiration measured (Figures 4C‐4E). To further confirm the impact of burn injury and ATGL ablation on mitochondrial activity, we assessed the activity of electron transport chain complexes by performing in‐gel activity assays. Following blue‐native polyacrylamide gel electrophoresis (BN‐PAGE) assays, the densitometric analysis demonstrated a significant increase in the activity of complex I and IV post‐burn, which was diminished in the burn AKO group (Figures 4F‐4J), suggesting that adipose‐specific ATGL ablation decreases mitochondrial activity. While no change was noted in ATP synthase, this could be a result of the increased uncoupling noted post‐burn. To further assess the impact of burn injury on mitochondrial content in iWAT, we assessed citrate synthase activity in the test groups. Although no change was observed in the burn AKO group, citrate synthase activity was elevated in burn WT group (Figure 4K). To assess the impact of ATGL ablation on iWAT browning post‐burn injury, we assessed the expression of UCP1, a key mediator of the iWAT browning response as well as other associated browning markers.^20^ As expected, based on the Seahorse analysis, IHC levels of UCP1 were elevated in burn WT mice and reduced in the AKO group (Figure 4L). The expression of key WAT browning modulators was further assessed via gene expression and protein expression. We observed that the expression of *Ucp1* was reduced in burn AKO mice in comparison to burn WT (Figures 4M and 4N). A similar impact was observed with the expression of the mitochondrial biogenesis marker *Pgc1α* and brown adipocyte biogenesis marker *Prdm16* (Figure 4M). Taken together, these findings indicate that adipose‐specific ATGL ablation diminishes the biogenesis and uncoupling of mitochondria associated with the pathological browning of iWAT 7 days after the burn injury.

### 2.4 Adipose‐specific ATGL deletion attenuates fatty liver development

**FIGURE 5:**
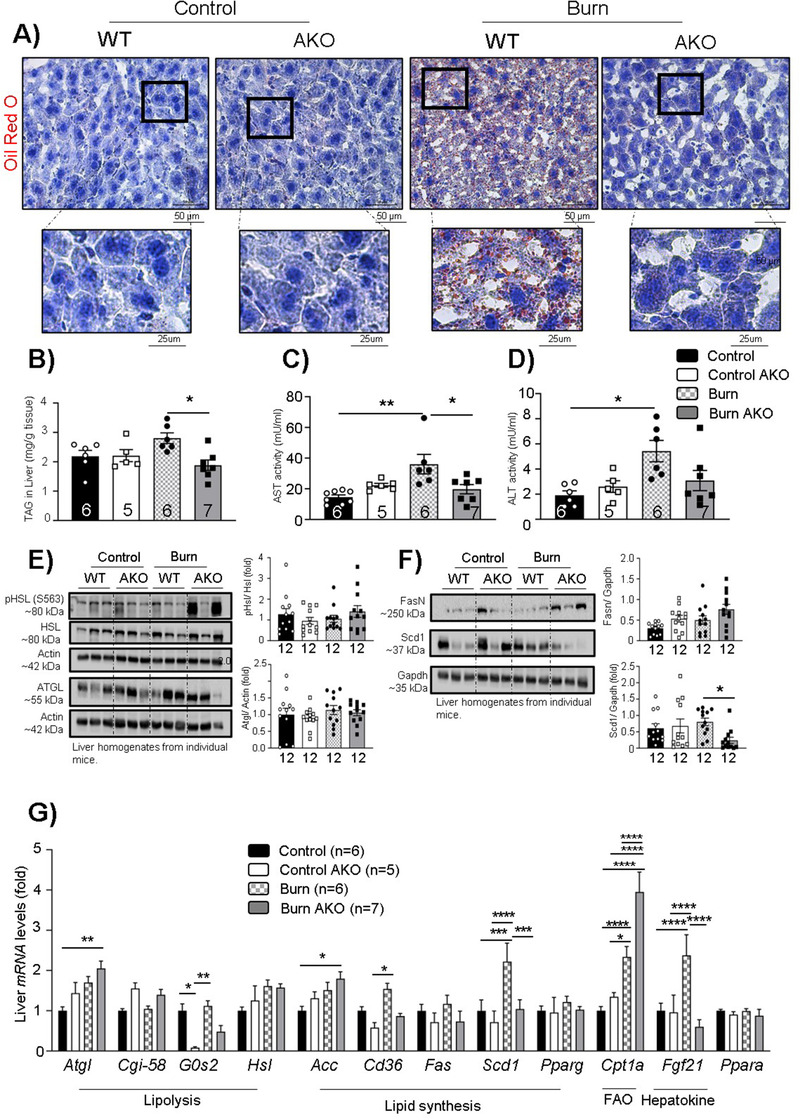
Adipose‐specific ATGL deletion rescues against fatty liver development post‐burn injury: Twelve‐week‐old ATGL floxed and knockout mice were treated with 30%TBSA injury and monitored daily for 7 days. (A) Oil Red O staining (zoom in and zoom out) for visualization of fat droplets in liver sections. (B) Tri‐acylglycerol (TAG) content in liver samples normalized to tissue weight. (C) Aspartate aminotransferase (AST) levels in serum samples. (D) Alanine aminotransferase (ALT) levels in serum samples. (E) Western blot analysis for the expression of targeted proteins (p‐HSL ser 563, HSL, ATGL, and actin) in the liver. (F) Western blot analysis and quantification of the expression of targeted proteins (Fas, Scd1, and gapdh) in the liver. (G) Quantitative PCR analysis of multiple genes in the liver. The results displayed are the average and SEM analyzed in the specified number of mice samples. Statistical significance was assessed using two‐way ANOVA as appropriate

Having established that adipose‐specific ATGL deletion reduces WAT lipolysis while decreasing mitochondrial activity, WAT browning and reducing systemic fat circulation post‐burn injury, we turned our focus to how this impacts the liver post‐burn injury. As measured by Oil Red O (ORO), burn WT mice showed enhanced deposition of fat droplets in the liver OCT (optimal cutting temperature compound) sections which was reduced in burn AKO mice (Figure 5A). Furthermore, burn AKO mice had reduced TAG levels in the liver in comparison to burn WT mice (Figure 5B). In addition, when we assessed liver function using classical biomarkers such as alanine aminotransferase (ALT) and aspartate aminotransferase (AST), burn WT mice showed enhanced ALT and AST levels while burn AKO mice demonstrated reduced AST and no impact on ALT levels in comparison to WT control mice (Figures 5C and 5D). These findings suggest that adipose‐specific ATGL ablation reduces the flow of FFA to the liver during chronic adrenergic activation, thus reducing fat deposition in the liver and protecting against hepatic metabolic dysfunction.

Next, we wanted to assess the impact of adipose‐specific ATGL deletion on lipolysis (ATGL and HSL), lipid synthesis (FASN, SCD1, ACC, and CD36), adipogenesis (PPARɣ), fatty acid oxidation (FAO) (CPT1a), and FGF21 hepatokine expression in the liver (Figures 5E‐5G). We did not see any impact of adipose‐specific ATGL ablation on the expression of lipolytic markers (ATGL and phospho‐HSL; Ser 563) (Figure 5E); however, hepatic *Atgl* gene expression was enhanced suggesting a compensatory effect to meet energy demand as there are reduced fats in circulation (Figure 5G). Despite the enhanced hepatic *Atgl* expression, no impact was observed on known ATGL coactivators such as *Cgi‐58* (Figure 5G) and inhibitors such as *G0s2* (Figure 5G).^21^ Furthermore, hepatic *Cd36* (cluster differentiation factor 36 or fatty acid transporter) and *Scd1* (stearoyl CoA desaturase‐1) gene expressions were elevated in burn WT mice, suggesting enhanced lipid synthesis due to enhanced systemic lipid circulation. This impact of burn injury was reverted in burn AKO mice (Figures 5F and 5G), implicating the indirect impact of adipose‐specific ATGL ablation on diminishing systemic lipid overload and thus, protecting the liver from enhanced ectopic fat deposition.

Moreover, we wanted to assess hepatic FFA oxidation and utilization by assessing the gene expression of carnitine O‐palmitoyltransferase 1 (*Cpt1a*), which is an indicator of FAO.^22^ *Cpt1a* expression was elevated in burn WT mice compared to control WT (Figure 5G), perhaps indicative of enhanced FFA substrate utilization to meet the high energy demand post‐burn injury. Intriguingly, *Cpt1a* expression was found higher in burn AKO mice as well in comparison to the burn WT group (Figure 5G), suggesting that adipose‐specific ATGL ablation indirectly further stimulates FAO in the liver. Moreover, hepatic *Fgf21* expression was found elevated in burn WT mice (Figure 5G), which is an indicator of liver‐WAT cross‐talk to elevate WAT browning in adaptive thermogenic conditions.^23^ However, hepatic FGF21 levels were comparable to WT controls in burn AKO mice (Figure 5G), suggesting that adipose‐specific ATGL ablation reduces FFA shuttling to the liver, consequently reducing FGF21 secretion from the liver. Taken together, these results point toward the protective impact of targeting adipose‐specific ATGL in preventing fatty liver development in hypermetabolic conditions.

### 2.5 Therapeutic targeting of ATGL reduces WAT lipolysis, browning, and hepatomegaly post‐burn injury

**FIGURE 6:**
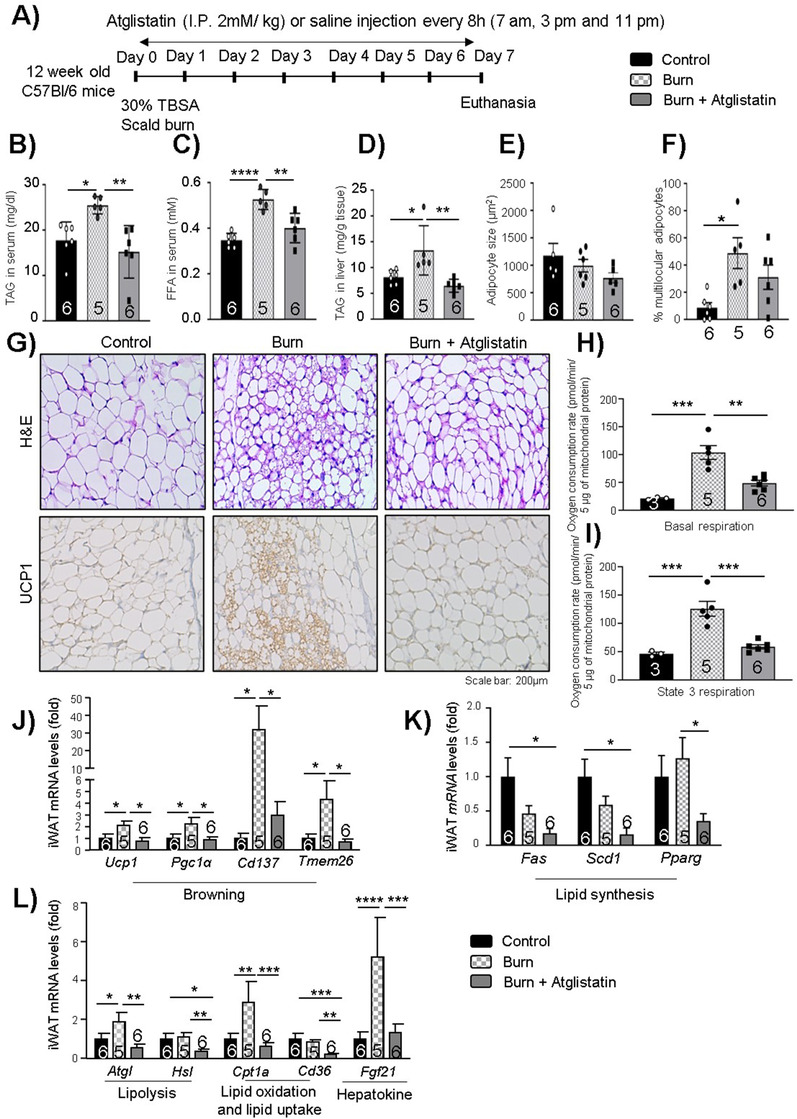
Therapeutic impact of atglistatin on iWAT post‐burn injury: Twelve‐week‐old C57BL/6 mice were treated with 30%TBSA injury and treated with Atglistatin (2 mmol/kg i.p.) starting at day 1 and monitored daily for 7 days. (A) Atglistatin treatment study plan. (B) Tri‐acylglycerol (TAG) content in serum samples. (C) Free fatty acid levels in serum samples. (D) TAG content in liver samples normalized to tissue weight. (E) Adipocyte size of adipocytes in iWAT. (F) Percentage of multilocular adipocytes normalized to total adipocytes. (G) Hematoxylin and eosin (H&E) staining and immunohistochemistry for UCP1 expression in WAT samples. (H and I) Basal, and state 3 respiration parameters were measured in isolated mitochondria using Seahorse assays. Quantitative PCR analysis of (J) browning markers (*Ucp1*, *Pgc1a*, *Cd137*, and *Tmem26*), (K) lipid synthesis markers (*Fas*, *Scd1*, and *Pparg*), and (L) lipolysis markers (*Atgl* and *Hsl*), lipid oxidation (*Cpt1a*), lipid uptake (*Cd36*), and hepatokine (*Fgf21*) markers in WAT. The results displayed are the average and SEM analyzed in the specified number of mice samples. Statistical significance was assessed using one‐way ANOVA tests as appropriate

Having established that adipose‐specific ATGL ablation is protective against enhanced WAT lipolysis and browning as well as fatty liver development, we wanted to assess the impact of therapeutic inhibition of ATGL using a tested ATGL inhibitor (atglistatin) in mice. C57BL/6 mice were divided into three groups (control WT, burn WT, and burn‐atglistatin treatment) and challenged with a 30% TBSA thermal injury. Atglistatin (2 mmol/kg i.p.) treatment was started on day 1 and continued every 8 h until day 7 post‐burn injury (Figure 6A). The dosage of atglistatin treatment every 8 h was chosen as per conditions defined elsewhere.^24^ As observed earlier, burn WT mice showed enhanced TAGs and FFAs in the systemic circulation, while burn atglistatin‐treated mice had reduced levels of these mediators (Figures 6B and 6C). A similar impact was observed on TAG deposition in the liver where atglistatin treatment reduced the accumulation of these moieties when compared to the non‐treated group (Figure 6D). Furthermore, when iWAT was stained with hematoxylin & eosin staining, and UCP1 IHC was performed, the thermally injured mice showed multilocular adipocytes and enhanced UCP1 expression, but no impact was observed in the burn atglistatin‐treated cohort (Figures 6E‐6G). The impact of atglistatin treatment on WAT lipid metabolism and browning was further assessed by gene expression analysis of lipolysis, lipid oxidation, fat uptake, and browning‐associated markers. As observed previously in burn AKO mice, atglistatin treatment also reduced mitochondrial activity and oxygen consumption for basal and state 3 respiration measured (Figures 6H and 6I). As expected, burn WT mice had elevated expression of browning markers (*Ucp1*, *Pgc1a*, *Cd137*, and *Tmem26*) in WAT, which was reduced in burn atglistatin‐treated mice (Figure 6J), suggesting a protective impact of atglistatin treatment against pathological WAT browning post‐burn injury. Furthermore, burn atglistatin‐treated mice showed reduced expression of lipid synthesis markers (*Fas, Scd1*, and *Pparg*) (Figure 6K). Indeed, *Atgl*, *Hsl*, and *Fgf21* gene expressions were elevated in the burn cohort with no treatment; however, burn atglistatin‐treated mice showed reduced expression of these markers (Figure 6L). Furthermore, thermally injured untreated mice showed enhanced expression of *Cpt1a* (Figure 6L), which is a key enzyme for FAO, suggesting enhanced fat breakdown as observed earlier in burn WT mice. Compared to burn WT, atglistatin treatment reduced *Cpt1a* expression, suggesting reduced FAO.

**FIGURE 7:**
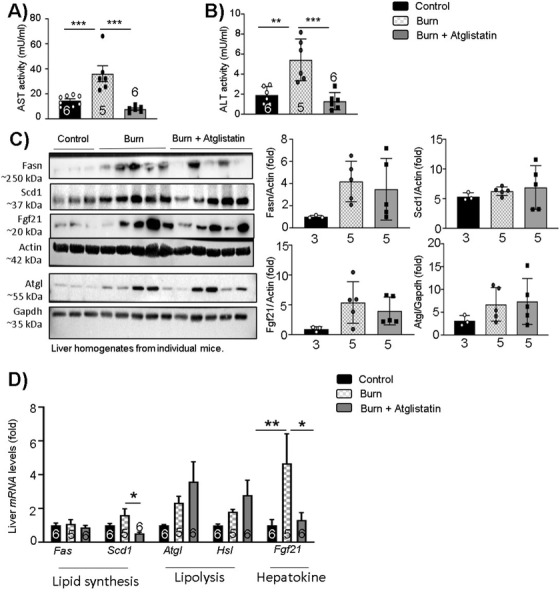
Therapeutic impact of atglistatin on liver post‐burn injury: Twelve‐week‐old C57BL/6 mice were treated with 30%TBSA injury and treated with atglistatin (2 mmol/kg i.p.) starting at day 1 and monitored daily for 7 days. (A) AST activity in serum samples. (B) ALT activity in serum samples. (C) Western blot analysis and quantification of target proteins in the liver. (D) Quantitative PCR analysis of lipid synthesis (*Fas* and *Scd1*), lipolysis (*Atgl* and *Hsl*), and hepatokine (*Fgf21*) markers in the liver. The results displayed are the average and SEM analyzed in the specified number of mice samples. Statistical significance was assessed using one‐way ANOVA tests as appropriate

The impact of atglistatin on liver function post‐burn injury was further assessed with ALT and AST measurements in serum and gene expression analysis of lipid synthesis and lipolytic signaling markers. As previously observed, AST and ALT activity was elevated in the burn WT group, while AST and ALT activity was repressed in burn atglistatin‐treated mice (Figures 7A and 7B). Furthermore, the untreated burn mice had enhanced expression of *Scd1* in the liver, a key lipid synthesis enzyme, which was found reduced in the treatment group (Figure 7D), suggesting reduced lipid synthesis in the liver with atglistatin treatment as a result of reduced substrate shuttling to the liver as observed in burn AKO mice. In agreement with KO findings, burn WT mice and burn atglistatin‐treated mice had no impact on hepatic expression of lipolytic (*Atgl* and *Hsl*) enzymes (Figures 7C and 7D). This observation is in line with the impact of atglistatin on the liver assessed by Schweiger et al where they showed that atglistatin treated mice liver had enhanced *Atgl* expression and reduced hepatosteatosis.^17^ It is not entirely clear why atglistatin fails to inhibit expression of ATGL in the liver. One possibility could be a compensatory effect by the liver to supply FFA and meet the high energy demand post‐burn, as circulating FFAs are lacking. Furthermore, burn WT mice had four folds higher expression of hepatokine (*Fgf21*) levels in comparison to control and burn atglistatin‐treated mice liver, suggesting that atglistatin treatment had a similar impact on *Fgf21* expression as observed in burn AKO mice (Figure 7D). Taken together, these findings suggest that targeting ATGL activity using atglistatin has a similar protective impact against lipolysis, pathological browning, and fatty liver complications as observed previously in burn AKO mice.

## 3 DISCUSSION

A severe burn injury induces a substantial and prolonged hypermetabolic response with accompanying complications such as hyperlipidemia, fatty liver development, and cardiac overload, complex challenges ultimately leading to poor outcomes in burn patients.^6^, ^25^, ^26^ While WAT lipolysis and browning may be a desirable phenomenon in other metabolic disorders such as obesity, these are major drivers of the adverse hypermetabolism and systemic consequences thereof post‐burn.^10^, ^27^, ^28^ WAT remodeling enhances circulating lipid levels and results in ectopic fat deposition in vital organs (such as liver, muscle, kidneys, and the heart) and thereby, affects crucial signaling pathways and organ function.^1^ In turn, severely burned patients are prone to developing fatty liver, insulin resistance, kidney, and heart failure in addition to poor wound healing and sepsis that profoundly increases the risk for morbidity and mortality.^25^ Targeting WAT lipolysis and browning have recently been demonstrated to be a promising approach to circumvent metabolic dysfunction in burn patients.^10^, ^12^, ^13^, ^27^ For instance, a recent study conducted in C57BL/6 mice challenged with severe burn injury has demonstrated the implications of elevated WAT lipolysis and browning in the progression of fatty liver development and metabolic dysfunction.^10^ Furthermore, treating thermally injured C57BL/6 mice with the lipolysis inhibitor acipimox resulted in reduced circulating fatty acids, attenuated WAT browning, and protected against fatty liver development.^13^ Acipimox, a niacin derivative, targets and reduces intracellular cyclic adenosine monophosphate substrate and indirectly, inhibits protein kinase A‐mediated HSL activation and activates lipogenesis.^13^ To that effect, niacin and derivatives thereof can also function by improving systemic NAD^+^ levels, subsequently restoring faulty processes such as autophagy and mitophagy. Moreover, a therapeutic agent given intraperitoneally is not a substitute for proper mechanistic studies. To that end the purpose of the current study was to determine if ATGL and lipolysis itself contributes to the pathological changes in adipose and the development of systemic dysfunction as a consequence of a severe burn injury. Our studies indicate that targeting ATGL, a rate‐limiting enzyme in the signaling cascade of lipolysis can be protective by reducing WAT lipolysis and fatty acids in circulation, which are the main drivers of WAT browning and fatty liver development and thus, prevent the associated complications of WAT breakdown.

In this study, we have shown that *Atgl*, *Ucp1*, and *Fgf21* expression levels are elevated in severe burn patients ≥ 7 days post‐burn. Furthermore, this is in line with our murine studies, where WAT levels of FGF21 appear to peak on day 7 with elevated expression of ATGL and UCP1 from day 7 onwards. These findings suggest the presence of a chronic response to burn injury with time‐dependent physiological changes to adipose tissue and a possible interplay between FGF21 circulation, adipose lipolysis, and thermogenesis. We noted a similar increase in hepatic FGF21 on day 7 post‐burn, suggesting a cross‐talk between the liver and adipose which likely enhances WAT browning. Indeed, previous studies under conditions of adaptive thermogenesis have demonstrated the physiological role of hepatic FGF21 in regulating thermogenic gene expression and the WAT browning response when exposed to cold exposure or β‐adrenergic stimulating compounds.^23^ Moreover, studies conducted in UCP1 KO mice challenged with severe thermal injury have shown the promising impact of reducing thermogenesis in the prevention of hepatic steatosis and metabolic dysfunction.^10^ Studies conducted in a UCP1/FGF21 double knockout (dKO) model have demonstrated a full reversal of obesity resistance in dKO mice by inhibiting the metabolic reprogramming of WAT genes responsible for enhanced lipid and oxidative metabolism.^29^ Our studies indicate the possible cross‐talk between the liver and WAT. However, whether the promotion of WAT browning occurs solely as a consequence of hepatic FGF21‐mediated activation, or whether this involves enhanced WAT lipolysis‐mediated substrate availability, or indeed alternate mechanisms, remains to be resolved. As FFAs are known inducers of hepatic FGF21 via PPARα activation,^30^ we propose a futile cycle of adipose lipolysis and FGF21 release which perpetuates the hypermetabolic response to burn injury. However, further studies are required in the FGF21 KO murine models to further confirm this evidence.

The studies conducted in adipose‐specific ATGL KO mice demonstrated that ATGL ablation decreases the loss of body and adipose mass while reducing hepatomegaly, key manifestations of the hypermetabolic response to thermal injury. Furthermore, circulating FFAs and TGs were found to be reduced in burn AKO mice, suggesting the preservation of fat mass in adipose tissue depots prevents systemic lipotoxicity. It is of interest to note that burn AKO mice had elevated phosphorylation of HSL at serine 563 in WAT, suggesting a physiological compensation for the lack of ATGL post‐burn. However, ATGL has 10‐fold more substrate specificity than HSL.^18^ Thus, targeting adipose ATGL reduces WAT lipolysis and limits intrinsic and extrinsic FFA availability. FFAs are a substrate for β‐oxidation and fuel for the WAT browning response.^31^, ^32^ Indeed, burn AKO mice showed reduced mitochondrial respiration, *Ucp1* expression, no change in citrate synthase activity, and a reduction of the mitochondrial biogenesis marker *Pgc1a*, thus limiting the thermogenic response in adipose following thermal injury. Moreover, *Fgf21* expression was found reduced in the WAT of burn AKO mice, indicating the possible impact of limiting FFA availability to the liver and thus, reducing FGF21 signaling in WAT and consequently WAT browning. However, further studies are required in a liver‐specific FGF21 knockout murine model to confirm this finding. Overall, these findings reaffirm the importance of targeting ATGL and associated FFA release in preventing pathological changes to the WAT.

Strikingly, our studies indicate that targeting adipose‐specific ATGL protects against the development of fatty liver post‐burn injury. The liver, as a central metabolic organ, is of great importance for the maintenance of glucose and lipid homeostasis in severely injured burn patients.^6^, ^25^, ^26^, ^33^ Adipose‐specific ATGL deletion restricts the FFA supply to the liver, reducing fat deposition in hepatocytes and thus, reduces lipid synthesis as assessed by decreased SCD1 expression. SCD1 is an important mediator of FA re‐esterification and lipid synthesis in WAT and the liver. Studies targeting whole‐body SCD1^34^ and specifically in WAT^35^ have shown great benefits on lipid handling and overall metabolic health. Furthermore, targeting SCD1 in WAT and the liver was insufficient in improving overall metabolic health and energy homeostasis in obesogenic conditions;^36^ however, it indicates a crucial cross‐talk between liver and adipose tissue to maintain energy homeostasis. Intriguingly, hepatic *Atgl* gene expression was elevated in the livers of burn AKO mice, suggesting that adipose‐specific ATGL deletion had no impact on hepatic ATGL expression and possibly that the liver is enhancing lipolysis to meet the enhanced energy requirement post‐burn injury. Moreover, *Cd36* gene expression was reduced, and *Cpt1a* expression was significantly increased in the liver of burn AKO mice. Cd36 is a fatty acid transporter protein responsible for the shuttling of fat in the liver. Studies in hepatocyte‐specific Cd36 KO mice have shown an attenuation in fatty liver development and improved insulin sensitivity in obese mice.^37^ Hepatic Cpt1a, on the other hand, is a crucial mediator for FAO, utilization and restrains systemic catabolism during starvation.^38^ Although *Cpt1a* expression was elevated in burn WT and burn AKO mice, *Cpt1a* expression was two times higher in burn AKO mice in comparison to burn WT mice livers, suggesting enhanced FFA utilization and reduced fatty liver development. These results are consistent with others that demonstrated in obese mice that adipose‐specific ATGL deletion protects against hepatosteatosis and high fat diet‐induced insulin resistance.^17^ However, we did not see any impact on insulin signaling in our model (data not shown). It might be due to the fact that these studies are conducted in lean mice, and prolonged time periods are not assessed.

Our results demonstrate that ATGL is a key mediator of adipose depot thermogenesis and breakdown via lipolysis, releasing FFAs that contribute to fatty liver and hepatic dysfunction. To further corroborate our findings, we administered the ATGL inhibitor atglistatin to thermally injured C57BL/6 mice. As expected, based on our KO studies, atglistatin treatment also resulted in reduced systemic FFAs and TAG deposition in the liver. Moreover, the expression of WAT lipolysis markers (*Atgl* and *Hsl*) was reduced in the treated cohort. Strikingly, *Ucp1* expression was also reduced in atglistatin‐treated WAT in addition to other browning‐associated markers such as *Cd137*, *Tmem26*, and mitochondrial biogenesis (*Pgc1α*), suggesting the therapeutic targeting of ATGL limits changes to WAT physiology. A similar impact was observed on the expression of *Cpt1a* and *Cd36*, suggesting reduced FA oxidation and uptake in WAT with atglistatin treatment. In addition, *Scd1* expression was also down‐regulated in the livers of the atglistatin‐treated cohort, while *Atgl* and *Hsl* expressions were upregulated as observed previously in the burn AKO group except for *Hsl*. Furthermore, hepatic and iWAT FGF21 levels were reduced in atglistatin‐treated burn mice. These results demonstrate the beneficial potential of targeting ATGL using atglistatin in burn patients to mitigate WAT lipolysis, pathological browning, and the systemic consequences thereof. However, pharmacological studies of atglistatin are limited to murine models as there is no equivalent drug available in humans at this juncture. Our studies indicate the potential therapeutic benefit of atglistatin for severe burn injuries. One possible means to test this hypothesis could be gene therapy against adipose‐tissue‐specific ATGL in hypermetabolic patients. Although human gene therapy has the potential for numerous medical benefits,^39^ the long‐term effects and implications of gene therapy are still being investigated.

Severe burns induce a chronic hypermetabolic response that contributes to poor outcomes via the catabolism of adipose depots and ectopic deposition of fat systemically. The results of this study point toward the beneficial impact of reducing adipose‐specific ATGL expression in hypermetabolic conditions to mitigate WAT lipolysis, pathological browning, and the development of liver dysfunction. Although targeting ATGL cannot replace the need for anti‐hyperglycemic agents in critically ill patients, combination therapy can reduce WAT lipolysis and the associated metabolic dysfunction. Our findings shed light on the mechanisms that contribute to physiological changes to WAT, such as the possibility of liver‐adipose cross‐talk mediated by FGF21 secretion from the liver and the role of ATGL in this process. However, further studies are required with respect to FGF21 to explore the mechanistic role of this moiety in WAT remodeling. We suggest that countering WAT lipolysis by specifically targeting ATGL would have therapeutic benefits in hypermetabolic patients and propose that the development of a human analog to atglistatin would improve outcomes by halting detrimental physiological changes to adipose.

### 3.1 Limitations of the study

Unfortunately, we only have access to blood samples as well as skin and adipose excised from the wound of patients. Therefore, we are not able to assess *Fgf21* expression in the liver which would provide important information to draw conclusions regarding liver‐WAT crosstalk post‐burn injury. Furthermore, we do not have access to a thermoneutral (30°C) room in our institution. All the animal studies performed in this study are carried out at ambient temperature (22‐23°C), whereas burn patients are often kept at temperatures ranging from 25 to 35°C to prevent hypothermia.^40^ As such, mice are expected to experience a basal level of cold‐induced thermogenesis,^14^ and warmer temperatures (30°C) would reduce adipose browning but likely not prevent it.^41^ Nevertheless, we are hoping to secure temperature‐controlled metabolic caging for future studies on burn‐induced thermogenesis. At higher temperatures, it is possible the catabolism of adipose tissues is less drastic, reducing the fat burden placed on the liver, although this remains to be explored.

## 4 MATERIALS AND METHODS

### 4.1 Patients

Burn patients admitted to the Ross Tilley Burn Centre (Sunnybrook hospital, Toronto, Canada) were consented preoperatively for tissue collection before undergoing surgery. For more information regarding burn patient demographics, refer to Table S1. For controls, we obtained fat from six non‐burn patients (controls) undergoing elective surgery.

### 4.2 Animal studies

Age‐matched adult male mice (10–12 weeks) with C57BL/6 background were used for animal studies. Mice were maintained in an ambient temperature‐controlled environment having 12 h day and night cycle with an adequate supply of chow diet and water. All animal study procedures were performed in at least three independent studies. Animal studies were approved by the animal ethics care committee of the Sunnybrook Health Sciences Center. Mice were anesthetized in isoflurane (3%–5%) anesthesia chamber and administered buprenorphine (0.1 mg/kg,intraperitoneal [i.p.] injection) for pain management. Mice had nearly 40% of their front (dorsal region) and back (ventral region) area shaved with electrical shaver. To avoid excessive dehydration, mice were injected with ∼ 1 cc of lactate ringer's solution subcutaneously along the spine (∼ 2 ml) and i.p. injected (100 μl) before the thermal injury. The full thickness 30% TBSA scald burns were achieved by exposing the dorsal region for 10 s and the ventral region for 2 s in a 98°C water bath to prevent vital organ damage. Burned mice were kept in sterile cages with food and water ad libitum. Control WT (shaved only) mice underwent similar experimental procedures except for the burn injury. Burned mice were scored twice daily by both the study investigator and certified veterinarians to minimize pain and distress. For animal studies, C57BL/6 male mice were ordered from Jackson laboratory, and ATGL^fl/fl^ and ATGL^cre/+^ mice (a generous gift from Dr. Thurl E. Harris) were inbred, and genotyping was performed to identify floxed only and cre positive to identify the WT and knockout mice, respectively. The atglistatin treatment group was injected intraperitoneally with atglistatin (2 mmol/kg in PBS containing 0.25% Cremophor EL pH‐7.1, Sigma‐Aldrich) and burn WT controls with the vehicle every 8 h with a freshly prepared solution as described.^24^

### 4.3 Mitochondrial isolation, respirometry, and in‐gel activity assays

Mitochondria from freshly‐excised iWAT were isolated via differential centrifugation as previously described.^42^, ^43^ BCA (bicinchoninic acid assay) assays was performed to estimate protein amount and mitochondria plated on a 96‐well plate for bioenergetics analysis using a Seahorse XF96 analyzer. The mitochondrial respiration in a coupled state (10 μg/well) was analyzed in mitochondrial assay buffer containing 10 mM succinate as a substrate with 2 μM rotenone as described.^12^ Seahorse XF Wave software permitted the grouping of respiration data from different mice groups into a single representative line curve after normalization to mitochondrial protein content. To confirm the respirometric findings, isolated mitochondria from iWAT were also subjected to BN‐PAGE followed by in‐gel activity assays. Sixty μg of protein was loaded per lane and electrophoresed under native conditions as described.^44^ Reaction mixtures for Complex I, II, IV, and ATP synthase were prepared according to previous protocols.^45^ Protein complex gels were imaged colorimetrically using a Chemidoc Imaging System (Bio‐Rad), and Coomassie Blue staining was used to ensure equal loading. The protein densitometry was analyzed using Fiji (Image J) software.

### 4.4 IHC

Adipose tissue (inguinal, epididymal, or brown) and liver tissue were immediately fixed in 10% formalin for 1–2 days and transferred to ethanol (70%) until paraffin embedding. For H&E or IHC, tissues were sectioned and stained respectively with H&E or desired antibodies (such as ATGL [cell signaling; 30A4; rabbit mAb], UCP1 [Sigma‐Aldrich; U6382; rabbit pAb] or FGF21 antibody [Abcam; ab64857; rabbit pAb]), followed by secondary anti‐rabbit horseradish peroxidase (HRP)‐labeled antibody and DAB (3,3’‐Diaminobenzidine) staining for visualization. Liver biopsies were briefly snap‐frozen in OCT using dry ice and maintained at −80°C before sectioning and staining with ORO stain. ORO staining was performed to stain fat droplets accumulated in the liver. Briefly, frozen OCT blocks were sectioned (10 μm thickness), fixed in formaldehyde solution, and stained with ORO stain (10 min, room temperature) followed by rinsing under tap water. After ORO stain, slides were stained using H&E stain for a minute, and rinsed with water before preserving in mounting medium. Imaging was performed using an LSM confocal microscope, Zeiss, Germany.

### 4.5 Adipocyte size measurement

WATs (iWAT and eWAT) were fixed in 10% neutral‐buffer formalin and processed for H&E staining. Once processed, images were captured using a Leica 2500 DM light microscope (Leica Microsystems, Concord ON, Canada) at ×20 magnification. Qualitative and quantitative analyses were conducted in both iWAT and eWAT by two blinded observers. In eWAT, adipocyte cell surface area was quantified from three sections of tissue per mouse (*n* = 5–6 mice/group). In iWAT, percent multilocularity was quantified by labeling unilocular and multilocular cells and calculating the percent of multilocular cells/total adipocytes in the field of view and taking the average from two to three sections of tissue per mouse (*n* = 5–6 mice/group). All quantifications were conducted using ImageJ software.^46^

### 4.6 Biochemical analysis – Western blot

Blood and desired tissues were isolated from mice and snapped frozen in liquid N_2_ and stored at –80 °C for later analysis. Samples were homogenised using bead rupture in ice‐cold RIPA lysis buffer (50 mM Tris‐cl pH 7.4, 150 mM NaCl, 1% NP40, 0.25% Na‐deoxycholate, 1 mM PMSF) supplemented with protease (Millipore, #20‐201) and phosphatase (Thermo Fisher Scientific, #A32957) inhibitors. Lysate protein quantity was determined using a BCA assay to normalize and resolve the protein samples using a 10% SDS‐PAGE analysis. After SDS‐PAGE, the resolved protein gels were transferred to nitrocellulose membranes using wet transfer electrophoresis. After transfer, the nitrocellulose membrane was stained and imaged with ponceau S stain to check for equal loading and protein transfer. After imaging, ponceau‐stained blots were washed with washing buffer (Tris‐buffered saline with Tween (TTBS)) for 5 min and blocked with 5% skim milk prepared in TTBS for 1 h at room temperature. After blocking, blots were washed for 5 min in TTBS and probed with the desired primary antibodies overnight in a cold (4°C) room on a rocker. Primary antibodies include ATGL (1:1000, cell signalling #2138), HSL (1:1000, Cell Signaling #4107), phosphor‐HSL‐serine 563 (1:1000, Cell Signaling #4139), UCP1 (1:1000, Abcam ab234430), Fas (1:2000, Cell Signaling #8023), SCD1 (1:1000, Cell Signaling #2794), actin (1:5000, Cell Signaling #3700), gapdh (1:2000, Cell Signaling #97166), and tubulin (1:2000, Cell Signaling #2148). After overnight incubation with the primary antibody, membranes were washed with TTBS (3x, 10 min), followed by incubation with 1:2000 HRP‐linked secondary antibody in 5% skim milk for 2 h. Following incubation and TTBS washes (3x, 10 min), the detection of desired proteins was achieved using a Chemi Doc Imaging system (Bio‐Rad) by applying a 1:1 mixture of the chemiluminescent substrate from bio‐rad. Densitometry was performed for protein quantification using Image J (Fiji) software.

### 4.7 Real‐time polymerase chain reaction

The total RNA was isolated from snap‐frozen tissues using RNA‐zol (Sigma‐Aldrich) as per the manufacturer's guidelines. Normalized RNA was reverse transcribed to complementary DNA using the Applied Biosystems cDNA reverse transcription kit. Quantitative real‐time quantitative polymerase chain reactions (qPCR) were performed using the applied biosystems step‐one plus real‐time PCR system. Primer sequences are available upon request.

### 4.8 ALT/AST determination

Circulating ALT (#700260) and AST (#701640) levels were determined using Cayman chemicals kits to assess the liver damage post‐burn injury as per the manufacturer's instructions.

### 4.9 TAG determination

Circulating serum TAG and liver TAG levels were determined using Cayman chemicals kits (#10010303) as per the supplier's provided protocol.

### 4.10 FFA determination

Circulating FFA levels were determined using a Wako diagnostics kit (999‐34691, 995–34791, 991–34891, 993–35191, 276–76491) as per the supplier's provided protocol.

### 4.11 Citrate synthase activity assay

Citrate synthase activity was determined using a Sigma Aldrich kit (#MAK193) as per the supplier's provided protocol.

### 4.12 Statistical analysis

The data in the study are presented as mean ± SEM and analyzed using GraphPad prism software. Statistical significance was assessed using a student's *t*‐test, one‐way ANOVA or two‐way ANOVA followed by Bonferroni posthoc tests as indicated. A significance value of *p* < 0.05 is indicated as a single asterisk, *p* < 0.01 as a double asterisk, *p* < 0.001 as a triple asterisk, and *p* < 0.0001 as quadruple asterisk as indicated.

## CONFLICT OF INTEREST

The authors have no conflict of interest to declare.

## AUTHOR CONTRIBUTIONS

Supreet Kaur designed and performed experiments as well as wrote the manuscript. Christopher Auger designed and performed experiments, analyzed data, contributed to scientific discussions, and wrote portions of the manuscript. Dalia Barayan, Priyal Shah, Anna Matveev, and Carly M. Knuth performed experiments and analyzed data. Thurl E. Harris provided the adipose‐specific mice line, analyzed experiments, and proofread the manuscript. Marc G. Jeschke directed the studies, wrote, and edited the manuscript.

## Supporting information

Table S1 Human burn patient demographics

## Data Availability

The data generated during the study to support the findings are available upon request from the corresponding author (Dr. Marc Jeschke) upon reasonable request.
