# Supplementary figures and images for: Adipose‐specific ATGL ablation reduces burn injury‐induced metabolic derangements in mice
Source: Clin Transl Med. 2021 Jun 6;11(6):e417. doi: 10.1002/ctm2.417 (PMC8181198; doi:10.1002/ctm2.417)

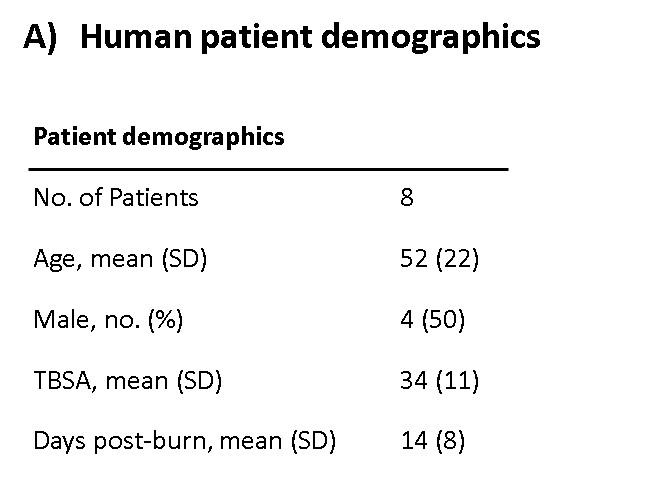

Supplement: Supplementary file 1 — Table S1 Human burn patient demographics [file CTM2-11-e417-s001.jpg]
